# Chemisorption of polysulfides through redox reactions with organic molecules for lithium–sulfur batteries

**DOI:** 10.1038/s41467-018-03116-z

**Published:** 2018-02-16

**Authors:** Ge Li, Xiaolei Wang, Min Ho Seo, Matthew Li, Lu Ma, Yifei Yuan, Tianpin Wu, Aiping Yu, Shun Wang, Jun Lu, Zhongwei Chen

**Affiliations:** 1 College of Chemistry and Materials Engineering, Wenzhou University, Wenzhou, Zhejiang, 325035 China; 2 Waterloo Institute for Nanotechnology, Department of Chemical Engineering, University of Waterloo, Waterloo, Ontario, N2L 3G1 Canada; 3 Department of Chemical and Materials Engineering, Concordia University, Montreal, Quebec, H3G 1M8 Canada; 4 Hydrogen & Fuel Cell Center for Industry, Academy and Laboratories, New & Renewable Energy Research Division, Korea Institute of Energy Research, Buan-gun, Jellabuk-do, 56332 Republic of Korea; 5 X-Ray Science Division, Argonne National Laboratory, Argonne, Illinois 60439 USA; 6 Chemical Sciences and Engineering Division, Argonne National Laboratory, Argonne, Illinois 60439 USA

## Abstract

Lithium–sulfur battery possesses high energy density but suffers from severe capacity fading due to the dissolution of lithium polysulfides. Novel design and mechanisms to encapsulate lithium polysulfides are greatly desired by high-performance lithium–sulfur batteries towards practical applications. Herein, we report a strategy of utilizing anthraquinone, a natural abundant organic molecule, to suppress dissolution and diffusion of polysulfides species through redox reactions during cycling. The keto groups of anthraquinone play a critical role in forming strong Lewis acid-based chemical bonding. This mechanism leads to a long cycling stability of sulfur-based electrodes. With a high sulfur content of ~73%, a low capacity decay of 0.019% per cycle for 300 cycles and retention of 81.7% over 500 cycles at 0.5 C rate can be achieved. This finding and understanding paves an alternative avenue for the future design of sulfur–based cathodes toward the practical application of lithium–sulfur batteries.

## Abstract (web summary)

Novel cathode design holds the key to enabling high performance lithium-sulfur batteries. Here the authors utilize anthraquinone to chemically stabilize polysulfides, revealing that the keto groups of anthraquinone play a critical role in forming strong Lewis acid-based chemical bonding.

## Introduction

Lithium**–**sulfur (Li**–**S) battery has been regarded particularly promising to take the place of the currently dominant lithium**–**ion batteries (LIBs)—which already reach their capacity limits—as the main power source in various applications ranging from portable electronics to electric**–**based vehicles (EVs)^1–3^. Li**–**S chemistry enables an extremely high energy (theoretical: 2600 W h kg^–1^ and practical: ~600 W h kg^–1^) of a rechargeable battery technology (~200 W h kg^–1^ of the state-of-the-art LIBs)^4,5^ with a low cost due to the natural abundance of S element^6^. However, the fast and dramatic performance decay precludes the broad implementation of Li–S battery in EVs^7,8^. The discharge intermediate lithium polysulfides species (Li_*x*_S_*n*_, 3 ≤ *n *≤ 8) dissolve in the liquid organic electrolyte, diffuse through the separator, and deposit on the lithium anode, predominantly resulting in severe self-discharge and decreased S utilization and Coulombic efficiency, namely the “redox shuttle effect”^9^. In addition, the intrinsic insulating nature of S and discharge product Li_2_S leads to inferior rechargeability and rate capability of Li–S battery^10^.

Over the past decades, tremendous effect has been devoted to circumventing the obstacles by constraining S within conductive matrix to alleviate polysulfides dissolution and migration during cycling and to improve the electrode kinetics^11,12^. The most common strategy involves infiltration or in situ growth of S into porous media, such as porous carbons^13^ and metal oxides^9^, polymers scaffolds, and MOFs/ZIFs^1,14^, forming S–host composite structure. These controllable porous architectures effectively retard the loss of active materials and prolong the cycle life of Li–S battery^15^. However, the simple spatial confinement cannot effectively function over extended period of time, owing to the weak intermolecular interaction between these hosts and polysulfides species, and up to an 80% of the volume variation of S during cycling. Another effective strategy to retain polysulfides involves engineering “sulfiphilic” surface with relatively strong interactions with polysulfide species^16^. For example, surface modification of carbon materials with oxygen/nitrogen/sulfur functional groups^16–20^ or the surface of hydroxides/oxides/sulfides/carbides^21–24^ can efficiently eliminate the polysulfide redox shuttle. However, lack of comprehensive understanding of such interactions from the molecular level and the elaborate preparation of the materials with “sulfiphilic” surface limit this strategy at the research stage. Moreover, significant amount of the transition metal hydroxides/oxides/sulfides has to be used to provide efficient “sulfiphilic” surface, which significantly reduces the total energy of the S electrodes. In this context, it is still highly desired to design novel trapping materials and to develop new mechanisms to enable enduring Li–S redox chemistry^25^.

In order to achieve high-performance S electrodes, three criteria should be satisfied to efficiently retain the polysulfides during cycling. (i) Soluble polysulfides reversibly bonded to or released from the electrode skeleton/host materials through reversible intermolecular Lewis acid-based bonding^26^. Such intermolecular bonding should occur not only on the surface but also within the whole host materials to minimize the portion of inactive components. (ii) The host materials should possess relatively high packing density with S to maintain both high specific and volumetric capacity of the S electrodes^27^. (iii) The host materials should be abundant or involve cost-effective preparation for future practical applications^28^.

Herein, we demonstrate, for the first time to the best of our knowledge, a novel strategy to develop highly stable S electrodes by utilizing commercially available or natural abundant organic small molecules with redox catalytic properties. Such organic molecules are capable of suppressing dissolution and diffusion of polysulfides species through redox reactions during cycling. Aromatic organic compound, anthraquinone (AQ), is selected as a representative to demonstrate this novel strategy, owing to its low cost and wide applications as redox catalyst and electrochemical energy materials^29–31^. We find that the keto groups of AQ play a critical role in confining polysulfides by forming strong Lewis acid-based chemical bonding. Moreover, AQ can be easily linked to graphitic carbon through π–π stacking^32^. A small portion of reduced graphene oxide (rGO) not only improves the conductivity but also further suppresses the polysulfides dissolution by forming intimate contact between AQ and rGO to promote long-cycling Li–S battery. Unlike other host materials, AQ is a type of organic molecule which has the potential to be highly distributed throughout the whole electrode and function. Furthermore, different from carbon-based materials or porous structures which often require large electrolyte/sulfur ratio (typically >15:1 µL mg^–1^), the small AQ molecules effectively increase the electrode density, which significantly improves both specific and volumetric energy density. With a high sulfur content of ~73%, the composite electrode delivers a high capacity and exceptional cycling stability with a capacity decay of 0.019% per cycle for 300 cycles and retention of 81.7% over 500 cycles at 0.5 C rate. Combined with density functional theory (DFT) calculations and in situ X-ray diffraction (XRD), such an interesting finding and understanding of redox reactions between lithium polysulfides and small catalytic organic compound along with the superior electrochemical performance paves a new avenue for the future design of S-based cathodes toward the practical application of high-performance Li–S batteries.

## Results

### Synthesis and characterization of S-AQ-G composites

**Fig. 1:**
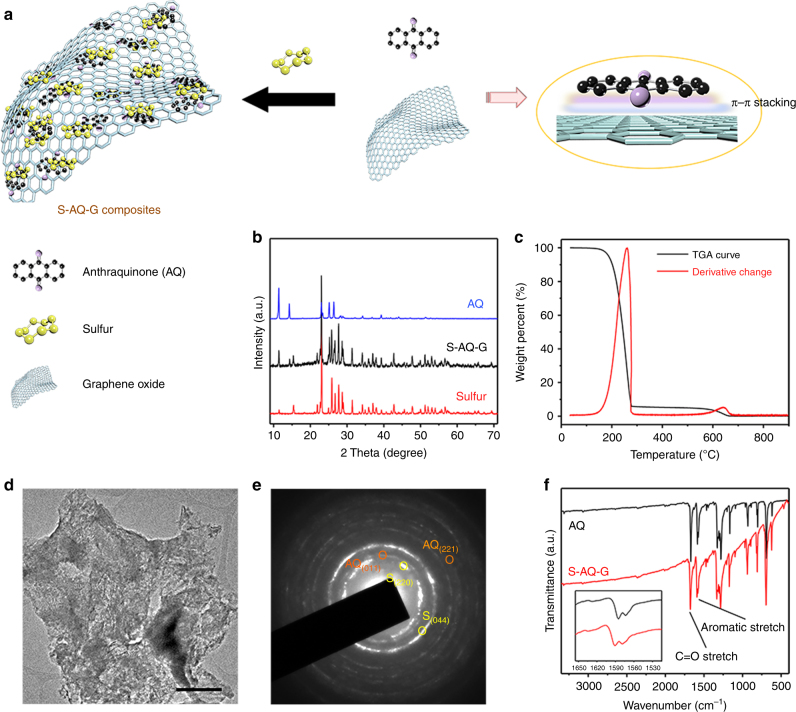
Synthetic route and characterizations of S-AQ-G composites. **a** Schematic illustration of the formation of S-AQ-G composites. **b** XRD patterns of pure S, pure AQ and S-AQ-G composites. **c** TGA and its corresponding derivative weight change of S-AQ-G composites. **d**,** e** TEM image and corresponding SAED of S-AQ-G composites (scale bar = 500 nm). **f** Comparison of the FTIR spectra of pure AQ and S-AQ-G composites

The as-synthesized composites (S-AQ-G) were achieved by attaching AQ to rGO through π–π stacking between the anthracene ring layers^32^, followed by the addition of S forming the homogeneous composites (Fig. 1a). Both AQ and S are highly crystalline according to the XRD patterns (Fig. 1b), which can be indexed to typical monoclinic (JCPDS No. 12–0851)^31^ and orthorhombic (JCPDS No. 08–0247)^33^ phases, respectively. The XRD peaks from both AQ and S are well maintained in the S-AQ-G composites without any unknown peaks observed from impurities, indicating that minimal or no reactions occur among the three components at the applied conditions. However, the existence of AQ and rGO with enhanced density of electron clouds effectively changes the status and activity of S^34,35^, which is revealed by the differential scanning calorimetry (DSC) result (Supplementary Fig. 1) with lower endothermic peak at around 120 ^o^C. Figure 1c shows the thermogravimetric analysis (TGA) curves. According to the results, the ratio of S is estimated to be ~73 wt% in the S-AQ-G composites. Both AQ and S are mixed uniformly, and distributed homogeneously on the surface of rGO nanosheets (Fig. 1d). Their highly crystalline nature is also evidenced by the selected area electron diffraction (SAED) spectrum shown in Fig. 1e, where corresponding crystal planes from both AQ and S can be observed clearly, further proving the evenly spreading of S and AQ with intimate contact to each other. To further analyze the S-AQ-G composites, Fourier transform infrared (FTIR) spectroscopy measurements were performed (Fig. 1d). The transmittance peak of AQ observed at around 1675 cm^–1^ can be related to the stretching modes of the carbonyl group (C = O), while peak at around 1590 cm^–1^ corresponds to the stretching modes of aromatic carbon rings^31^. For the S-AQ-G composites, no obvious new peaks—especially the peak from S–O stretching—can be found, implying all the components are in their original forms, which is consistent with the XRD and DSC observations. However, it is interesting that the peaks from AQ are found to shift toward the high-wavenumber region compared with those of pure AQ, which is mainly attributed to the π–π interactions between the Quinone molecules and the graphitic carbon surface^36^. The morphology and nanostructure of the S-AQ-G composites were characterized using transmission electron microscopy (TEM) measurements.

### Interaction between lithium polysulfides and AQ molecules

**Fig. 2:**
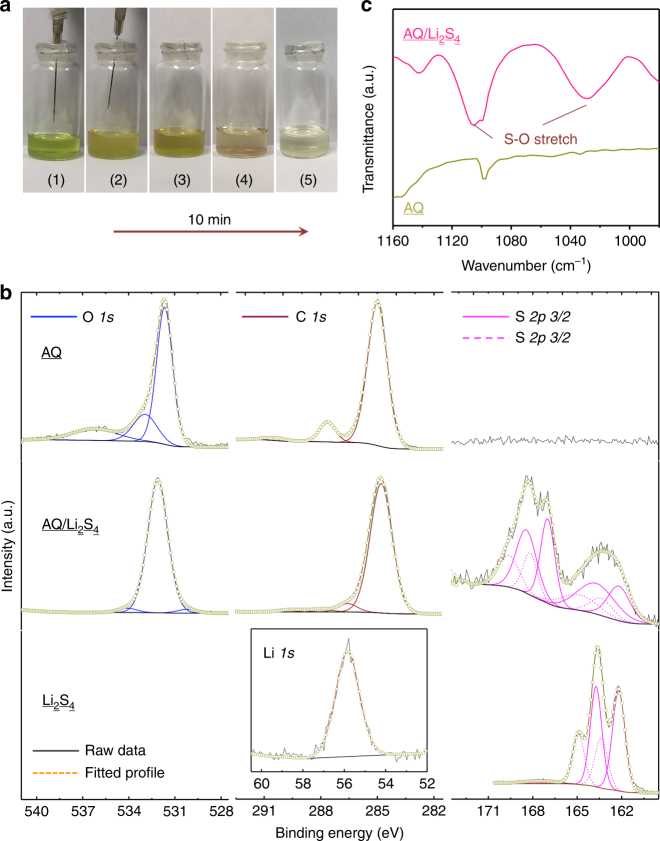
Analysis of the interactions between Li_2_S_4_ and AQ molecule. **a** Digital pictures showing the fast redox reaction between AQ and Li_2_S_4_: (1) before AQ injection, (2) during AQ injection, (3) after AQ injection, (4) after 5 min, and (5) after 10 min. **b** Comparison of XPS core-leveled spectra of elements O, C, and S in pure AQ, pure Li_2_S_4_, and recovered solid (AQ/Li_2_S_4_) from their mixed solutions. **c** Comparison of the FTIR spectra of pure AQ and AQ/Li_2_S_4_ composites

To analyze how AQ efficiently stabilizes S-based cathodes during a discharge process, we experimentally investigate the interactions between AQ molecules and lithium polysulfides. A representative lithium polysulfide species Li_2_S_4_ was synthesized according to the literature^37,38^ and dissolved in tetrahyrdofuran (THF) forming a transparent light-yellow solution. As shown in Fig. 2a, evidently, the injection of AQ@THF dispersion renders the Li_2_S_4_ solution colorless immediately, and the precipitation of light brown solid occurred shortly, implying either a strong adsorption capability of AQ or a chemical reaction between the two components. To further understand the strong interactions, X-ray photoelectron spectroscopy (XPS) was carried out on pure AQ, pure Li_2_S_4_, and the recovered solid (AQ/Li_2_S_4_) from their mixed solutions. Figure 2b compares the core-leveled spectra of element C, O, and S, where clearly, AQ is consist of only element C and O, while Li_2_S_4_ contains elements S and Li as well (Fig. 2b, inset). The Li_2_S_4_ shows two pairs of S doublets at 162.20/163.38 eV and 163.73/164.91 eV with an atom ratio of ~1:1 (Supplementary Fig. 2), corresponding to the terminal (S_*T*_^–1^) and bridge (S_*B*_^0^) S atoms, respectively, which is consistent with the typical linear structure of Li_2_S_4_ molecule^37^. By comparison, the S peaks from AQ/Li_2_S_4_ show a significant shift to the higher energy region, suggesting the oxidation state of S atoms increases. On the one hand, the molar ratio of S_*T*_^–1^ and S_*B*_^0^ decreases to 2:3 (Supplementary Fig. 3), implying the strong oxidation of terminal S atoms. On the other hand, the significant contribution between 166 and 172 eV is ascribed to the S = O or S–O groups^39^, formed by the redox reactions between Li_2_S_4_ and chemically active oxygen containing component. Obviously, the oxygen from C = O group in AQ molecule with high oxidative ability can promote this oxidation of Li_2_S_4_^32^. Such a redox reaction can further be revealed by the peak changes of C 1*s*, where C = O peak disappears while C–O peak arises^40^. Accordingly, the typical peak for C = O also vanishes in O 1*s* spectrum^41^. The formation of S–O and S = O chemically bonding is also supported by FTIR spectrum of AQ/Li_2_S_4_ (Fig. 2c), where S–O stretching modes can be found at 1030 and 1106 cm^–1^, respectively^42^. In a typical reaction, the Li_2_S_4_ molecule (or other polysulfides species) undergoes an attack of C = O group in AQ molecule due to its susceptibility to nucleophilic attack^43^, which results in the break of C = O double bond and formation of S–O interactions. Moreover, the delocalization of the electron structure of AQ molecule is also confirmed by the slight down shift of C 1*s* peak, accompanied by the up shift of O 1*s* peak, which is mainly due to the change of conjugate extended π bond.

**Fig. 3:**
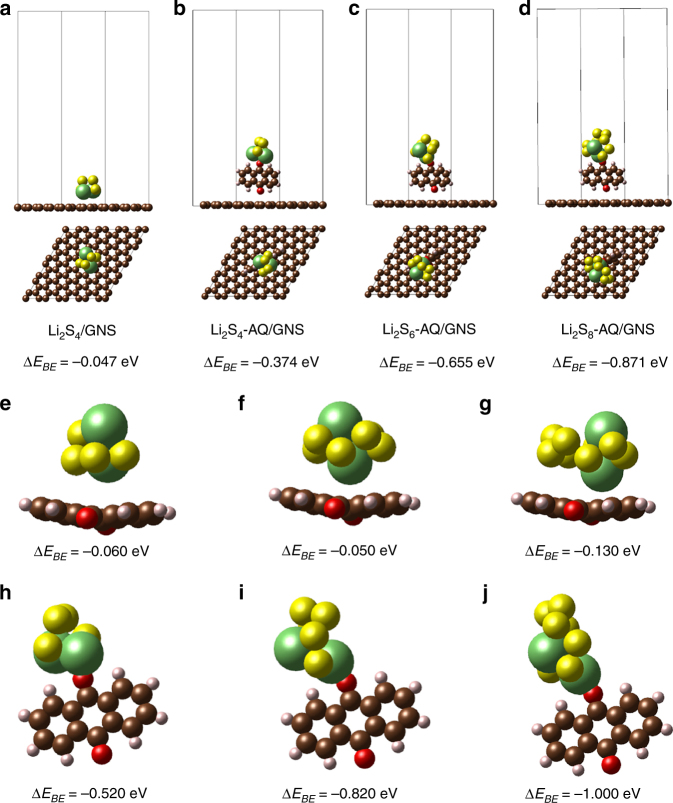
Simulation of lithium polysulfides adsorption by AQ. Atomic conformations and binding energies of **a** Li_2_S_4_ adsorbed by graphene; **b** Li_2_S_4_, **c** Li_2_S_6_, and **d** Li_2_S_8_ by AQ on the surface of graphene. Different atomic configuration and corresponding binding energies of lithium polysulfides with AQ molecule. Binding energies of Li_2_S_4_, Li_2_S_6_, and Li_2_S_8_ adsorbed by AQ molecule at oxygen site **e**, **h**, **f**, and on the plane **i**, **g**, **j** of AQ

The ab initio computational study further reveals the enhanced interaction between Li_2_S_n_ (4 ≤ *n* ≤ 8) and AQ on rGO. Graphene (Gr) was used in the designed model for simplicity purpose. In order to describe this interaction, the binding energies, *E*_*BE*_ were defined for interaction between Li_2_S_*n*_ and AQ, and between Li_2_S_*n*_ and AQ/Gr, as follows:

1 $$E_{BE} = E_{Li_2S_n/AQ} - E_{Li_2S_n} - E_{AQ}$$

2 $$E_{BE} = E_{Li_2S_n/AQ/Gr} - E_{Li_2S_n} - E_{AQ/Gr}$$

where *E*_*Li2Sn*_, *E*_*AQ*_, *E*_*Li2Sn/AQ*_, and *E*_*AQ/Gr*_ are the total energies of Li_2_S_*n*_ (4 ≤ *n* ≤ 8), AQ, Li_2_S_*n*_ with AQ and Li_2_S_n_ adsorbed on the AQ/Gr, respectively. The XRD patterns theoretically calculated from ICSD database for both AQ and sulfur (S_8_) align very well with the experimental results (Supplementary Fig. 4). It should be noted that in our DFT calculation, the binding of single molecule AQ on Gr is favored only if the AQ molecule is vertically located on Gr surface with a (4√3 × 4√3) unit cell (Supplementary Fig. 5a), where a negative binding energy of –0.037 eV can be achieved. Likewise, monoclinic crystal structure of AQs is favorably adsorbed vertically on Gr with a calculated binding energy of –0.026 eV (Supplementary Fig. 5b). However, the Li_2_S_n_ molecules are strongly adsorbed by AQ on Gr. As shown in Fig. 3a, without AQ molecule, the Li_2_S_4_ molecule can be spontaneously bonded to Gr only at a low coverage, with an extremely weak binding energy of –0.047 eV. By comparison, the Li_2_S_4_ molecule is strongly interacted with the AQ molecule on Gr with a significantly higher binding energy of –0.374 eV, almost an order of magnitude higher than that without AQ molecule (Fig. 3b). Similarly, other polysulfides species also exhibit strong adsorption with AQ on Gr. A binding energy of –0.655 eV and –0.871 eV can be achieved for Li_2_S_6_ (Fig. 3c) and Li_2_S_8_ (Fig. 3d), respectively.

In addition, polysulfide species are not adsorbed by AQ molecule simply through molecular interactions. We found that different configurations of polysulfide with AQ molecules lead to different binding effect. Figure 3 further compares the binding energies of polysulfide species (Li_2_S_4_ (Fig. 3e) and (Fig. 3h), Li_2_S_6_ (Fig. 3f) and (Fig. 3i), and Li_2_S_8_ (Fig. 3g) and (Fig. 3j)) adsorbed by AQ molecule at the oxygen site and on the plane. Obviously, Li_2_S_*n*_ is favorably adsorbed at oxygen site with much higher binding energy (–0.52 eV for Li_2_S_4_, –0.82 eV for Li_2_S_6_, and –1.00 eV for Li_2_S_8_). Such relatively high binding energies imply the formation of strong chemical bonds between polysulfide species and AQ molecule at the keto group, which is consistent with the XPS observation and our corresponding analysis mentioned above. It should be noted that since these binding energy values are in the mid-range of chemical bonds intensities, it implies that the redox reaction between polysulfide species and the AQ molecules are reversible.

### Electrochemical properties of S-AQ-G composites

**Fig. 4:**
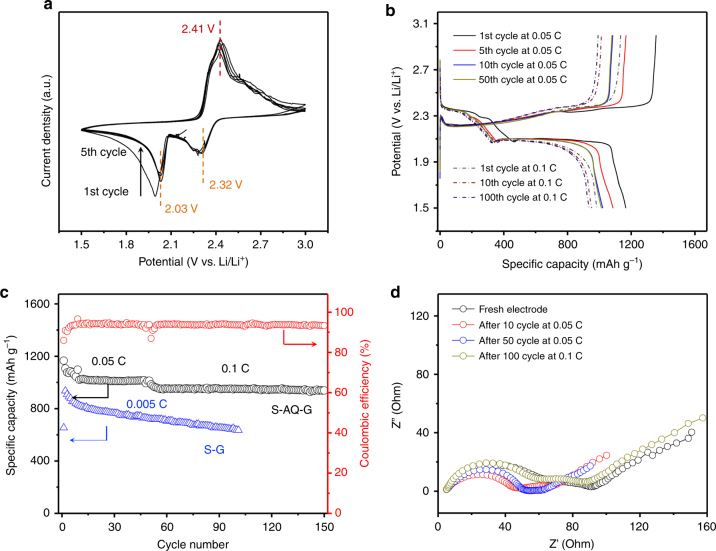
Improved electrochemical properties of S-AQ-G composites. **a** CV curves of S-AQ-G composites for the initial 5 cycles at a scan rate of 0.1 mV s^–1^. **b** Galvanostatic charge-discharge profiles of S-AQ-G composite electrode for various cycles at a current density of 0.05 and 0.1 C, respectively. **c** Corresponding capacity dependence on cycle number. **d** Nyquist plots of S-AQ-G composite electrode at different status

Obviously, such strong interactions between lithium polysulfide species with AQ on rGO and the redox reactions of Li_2_S_n_ (4 ≤ *n* ≤ 8) with AQ would provide the key factor in enhancing the cycling stability of S electrodes. The electrochemical behavior of the S-AQ-G composites was firstly investigated by cyclic voltammetry using CR2016 coin-typed cells with lithium metal as the counter electrode and 1.0 M lithium *bis*(-trifluoromethanesulphonyl)imide (LITFSI) in dioxolane/dimethoxyethane (DOL/DME; v/v = 1:1 with 2 wt% LiNO_3_) as the electrolyte. Figure 4a shows the typical cyclic voltammogram (CV) curves of the S-AQ-G composite electrode at a scan rate of 0.1 mV s^–1^ for the first 5 cycles within a voltage window of 3.0 to 1.5 V, where characteristics for electrochemical reaction of sulfur with lithium are observed^44,45^. Two major reduction peaks can be found at around 2.32 and 2.03 V during the cathodic scans, corresponding to the transformation of pristine S (cyclo–S_8_ for the initial cycle and Li_2_S_8_ for the following cycles) to long-chain lithium polysulfides (Li_2_S_*n*_, 4 ≤ *n* < 8), and the subsequent decomposition of long-chain polysulfides forming Li_2_S_2_ and/or Li_2_S, respectively^46^. However, upon closer analysis the first reduction peak appears to be composed of two peaks. We believe the double peak corresponds to first the reduction of S_8_ to polysulfide and its subsequent reaction with AQ which complements our XPS observation and analysis. During the anodic scan, the CV curves exhibit only one intense oxidation peak positioned around 2.41 V, attributing to the relatively slow kinetics of inverse polysulfides conversion process^47^. After the initial activation scan, no obvious changes occur for the subsequent cycles in terms of peak positions and intensities, indicating the reversible redox reactions of the S-AQ-G composites and cycling stability of the electrode. Figure 4b shows the typical galvanostatic charge–discharge profiles of S-AQ-G composite electrode for different cycles at 0.05 and 0.1 C, respectively (1 C = 1672 mA h g^–1^). Two distinct discharge and one charge plateaus are clearly shown for all profiles, aligning well with the CV observations^48,49^. The S-AQ-G composite electrode delivers an initial specific capacity of 1166 mA h g^–1^ at 0.05 C with the Coulombic efficiency of 86.1%, corresponding to 70.0% of the theoretical capacity. It should be noted that the pure AQ is electrochemically active but possesses an extremely low capacity and conductivity in the same system (Supplementary Fig. 6), and thus does not contribute significantly to the whole capacity.

Figure 4c displays the corresponding capacity dependence on cycling at 0.05 C for 50 cycles and 0.1 C for 100 cycles. The composite electrode shows a slight capacity decay for the initial equilibrium cycles, and remain stable with a discharge capacity of 1013 mA h g^–1^ after 50 cycles at 0.05 C. Moreover, a reversible discharge capacity of 938 mA h g^–1^ can be obtained after 100 cycles at 0.1 C, corresponding to a capacity retention of 95.2%. These behaviors indicate an excellent cycling stability of the S-AQ-G composites. By comparison, the S-G composite electrode shows a dramatic capacity decay with a much lower capacity (Supplementary Fig. 7), due to the fast loss of active materials. Although S-AQ-G appears to be quite stable, we should point out that the Coulombic efficiency remains at around 90% during cycling, which is a little lower than most of carbon–sulfur composites^50,51^, though LiNO_3_ was added to improve the efficiency. We believe this is mainly due to the electrochemical activity of AQ molecules, which gives negative effects on the Coulombic efficiency. Nevertheless, further investigation on this phenomenon is required^52^. The outstanding cycling stability was further supported by the electrochemical impedance spectroscopy measurements. Figure 4d compares the typical Nyquist plots of the S-AQ-G composites at various cycling status. The shape and size of the semicircle and Warburg tail remain with a small electrode series resistance, revealing a highly stable composite electrode^53^. It is worth mentioning that the typical S mass loading for each electrode is around 1.8 mg cm^–2^, which corresponds to a high areal capacity of ~2.0 mA h cm^–2^ at 0.05 C rate.

In order to correlate the electrochemical behavior of S-AQ-G composites with the proposed mechanism, the normalized CV curve of S-AQ-G composites was compared with that of S-G electrode (Supplementary Fig. 8a) at a scan rate of 0.1 mV s^–1^. Obviously, the redox peaks of S-G composites are deformed and widened, suggesting the sluggish kinetics. Moreover, both cathodic peaks of the S-AQ-G composites are located at higher voltage region with significantly reduced potential gap than those of the S-G counterpart, indicating higher reduction potential with suppressed electrochemical polarization for the composite electrode^54^. Likewise, lower oxidation potential is also an indication for efficient electrochemical system with improved electrode kinetics (Supplementary Fig. 8b). Although both S and AQ suffer from their intrinsically poor conductivity, the well-distributed S and subsequent polysulfides during cycling on AQ molecules and AQ/G composite sheets become more active due to not only the redox reactions with AQ but also the change of the electron structure induced by AQ/G catalytic effect^55^. Comparison of the onset potentials of S-AQ-G and S-G composite electrodes (Supplementary Fig. 8c) further confirms the accelerated redox processes of S-AQ-G. The S-G composites show an onset potential of 2.26 V for the anodic scan and 2.42 and 2.06 V for the cathodic scan. By comparison, the S-AQ-G composites exhibit similar oxidation onset potentials of 2.27 V, but higher reduction onset potentials of 2.43 and 2.09 V, respectively, indicating much improved kinetics with lower polarization.

### In situ XRD analysis

**Fig. 5:**
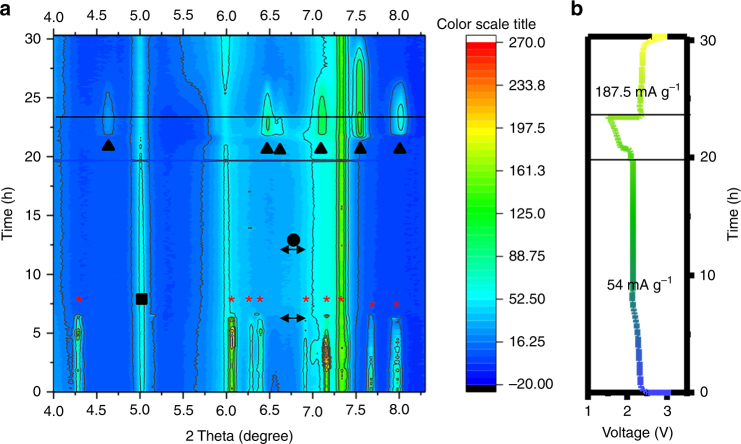
In situ XRD analysis of the interactions during cycling. **a** XRD intensity heat map from 4 to 8.5^o^ of a 2.4 mg cm^–2^ cell’s first cycle discharge at 54 mA g^–1^ and charge at 187.5 mA g^–1^, where triangles = Li_2_S, square = AQ, asterisk = sulfur, and circle = potentially polysulfides 2θ. **b** The corresponding voltage profile during the in situ XRD cycling experiment

To further investigate the mechanism of AQ, in situ XRD was performed with the results shown in Fig. 5a (XRD heat map) and Fig. 5b (voltage profile during XRD testing). It is apparent that almost all the sulfur peaks (labeled with asterisks) disappeared after discharge. This corroborates well with the observed high discharge first plateau discharge capacity of ~420 mA h g^–1^ from the cell’s voltage profile. Near the end of discharge, new peaks (labelled with triangles) are found, which we associate with Li_2_S.  As expected, most of these peaks disappear early in the charging cycle and mostly depleted at the end of charge. Interestingly, sulfur peaks are never returned to its previous intensity. Considering the XPS analysis, we believe sulfur or polysulfides have reacted with AQ molecules and can no longer form large enough sulfur domains for yielding any appreciable XRD peaks. Even more intriguing is the evolution sequence in the broad peak cluster from 2θ = 6.7^o^ to 6.9^o^ (indicated with a double-headed arrow) which increases in intensity from 7.5 (right before end of first plateau) to 12.5 h (well into the second plateau) followed by a decrease in intensity from 12.5 to18 h. The unique sequence of this observation suggests two possible phenomena. The intensity increase from 6.7 – 6.9^o^ is due to the broadening of the 6.9^o^ S_8_ peak and the reestablishment of sulfur peaks in the form of small crystals domains (broad peak). However, since the increased intensity proceeds well into the second plateau which is well known to involve only the transition of polysulfides to Li_2_S it is doubtful that appreciable regeneration of S_8_ nano crystal domains is occurring. Therefore, we believe the extended shoulder at 6.7^o^–6.9^o^ (shown on Fig. 5 by a double-headed arrow) is related to the generation of polysulfide species and is in actuality, a weak peak of its own. If analyzed from this perspective, this peak begins to brighten right from the beginning of discharge and begins to disappear near the middle of the second plateau. This is in good agreement with the discharge mechanism since higher order polysulfides also increase from the beginning and disappear towards the end of discharge. If we combined our XPS analysis with this unusual sequence of XRD peak evolution, we suspect our proposed reaction of AQ with polysulfide brought certain higher order polysulfides out of solvation and produced small polysulfide crystal domains. A similar phenomenon has been observed by Villevieille and colleagues^56^, where polysulfide adsorption onto silica yielded appreciable XRD peaks^57^. Another interesting phenomenon is the broadening of S peak at 2θ = 6 to almost 5.5 after charge. We believe this can mainly be attributed to the sulfur particle size (assuming they are similar to crystal domain size) becoming smaller after the first cycle. This could be further evidenced by the fact that the well-distributed AQ was able to catch the dissolved lithium polysulfides and re-distribute the sulfur into smaller particles.

Finally, it is also worth noting that sulfur peaks at ~4.3^o^, ~6.4^o^, and ~7.2^o^ developed sharper peaks (more yellow) as discharge progresses at 5 h which corresponds to 270 mA h g^–1^ or 2.3 V (prior to the second plateau). By the Scherrer equation, this implies that certain sulfur domains are increasing in size as the cell discharges and then disappears again as the cycle proceeds. These peaks are most likely an indication of the plane of sulfur that is especially abundant during disproportionate reactions (S_8_ re-precipitation). Disproportionation reaction can occur either in or out of the cathode depending on how efficient the PS is retained by the electrode. Since these peaks can completely disappear, it is implied that the electrode is able to efficiently retain polysulfide within the cathode and no irreversible sulfur deposition (via disproportionation reaction) are occurring out of the cathode.

### Electrochemical performance of Li–S battery

**Fig. 6:**
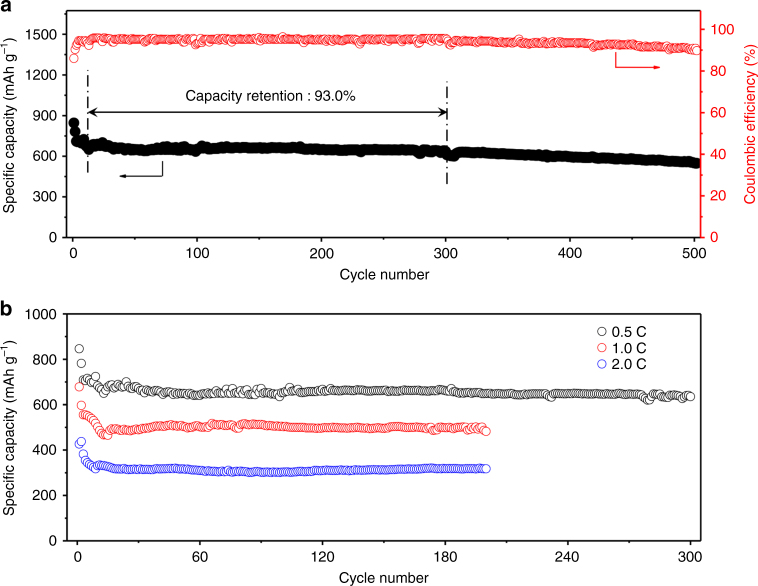
Electrochemical performance of Li*–*S battery. **a** Long-term cycling performance of Li**–**S battery cell at 0.5 C for 500 cycles. **b** Comparison of the cycling stability at various C rates

Such redox reactions between lithium polysulfides and AQ molecules provide high feasibility for highly stable S-based cathode towards the practical application of Li–S batteries. Figure 6a displays the long-term cycling performance of S-AQ-G composites at 0.5 C rate, corresponding to a charge/discharge of ~45 min. An initial discharge and charge capacity of 846 and 985 mA h g^–1^ can be delivered, with a Coulombic efficiency of 85.9%. After 10 equilibrium cycles, the S-AQ-G composites show an extremely stable and reversible capacity without obvious performance decay for almost 300 cycles. A discharge capacity of 635 mA h g^–1^ can be maintained, which corresponds to a capacity decay of only 0.019% per cycle (calculated based on the capacity for 11th cycle). Although a slight capacity fading occurs between 300 and 500 cycles along with the efficiency decay, which is obviously attributed to the gradually unavoidable Shuttle effect, a discharge capacity of 550 mA h g^–1^ can be obtained, with a high capacity retention of 81.7%. It is worth mentioning that the specific capacity of S-AQ-G is slightly lower than that of some of the C/S composite materials, which is mainly due to the lack of a large amount of conductive agent (less than 5% of graphene in S-AQ-G composites) and poor conductivity of both S and AQ. Remarkably, such excellent cycling stability can also be achieved at high current densities. As shown in Fig. 6b, the S-AQ-G composites exhibit a discharge capacity of 482 and 318 mA h g^–1^ at 1.0 and 2.0 C rate after 200 cycles, respectively, corresponding to a capacity retention of 71.1 and 74.4% (calculated based on the initial capacities).

As we mentioned, although some of the S-based composites have been reported with similar or even better electrochemical cycling stability, elaborate syntheses are often ineluctable in the construction of composite nanoarchitecture, which results in low mass loading of active materials and decreased energy density of the cathode^58–63^. By comparison, utilizing commercialized organic compounds such as AQ and its derivatives is completely compatible with current battery fabrication technologies, significantly lowering the manufacturing cost without sacrificing the electrochemical performance^64^.

## Discussion

In summary, we have demonstrated an innovative strategy for the efficient protection of lithium polysulfides towards high-performance Li–S batteries by enabling redox reactions of small organic compounds. Commercialized AQ molecule has been investigated as the effective polysulfides-confining agent, which possesses well-defined and unique molecular structure with high reactivity with soluble polysulfides, enabling extremely stable S-based cathodes. This novel strategy along with the understanding of the redox mechanism is compatible with current battery fabrication technologies, which brings high-performance Li–S batteries a step closer to the future practical application.

## Methods

### Synthesis of S-AQ-G composites

Graphene oxide (GO) was prepared from powdered flake graphite by a modified Hummers’ method. Sulfur (S) and anthraquinone (AQ) were purchased form Sigma-Aldrich and used as received. In a typical synthesis, GO (111 mg) and AQ (250 mg) were homogeneously mixed and vacuum-sealed in an ampoule before heated at 160 ^o^C for 12 h at a ramping rate of 1 ^o^C min^–1^. S (750 mg) was added to the ampoule and vacuum-sealed again before heated at 160 ^o^C for another 24 h at a ramping rate of 1 ^o^C min^–1^. The S-AQ-G was collected after cooling. The S-G composites were prepared directly by vacuum-sealing S (750 mg) and GO (111 mg) in an ampoule and heated at the same condition.

### Synthesis of Li_2_S_4_ and AQ/Li_2_S_4_

The Li_2_S_4_ was synthesized according to the literature^39^. In a typical synthesis, S was fully dissolved in Super-Hydride Solution (1.0 M lithium triethylborohydride in THF) in a molar ratio of 2:1. The resulting solution was dried under vacuum, and a yellow powder precipitate was obtained. The yellow powder was washed by toluene and isolated by centrifugation for several times to obtain the Li_2_S_4_ powder. The Li_2_S_4_ was dissolved in THF forming a transparent light green solution, while AQ was dispersed in THF. The injection of AQ@THF dispersion renders the Li_2_S_4_ solution colorless immediately, and the precipitation of light brown solid shortly. The AQ/Li_2_S_4_ was collected from the recovered solid.

### Morphology and structure characterization

XRD measurements were performed on a Rigaku Miniflex 600×-Ray Diffractometer (40 kV, 25 mA, Cu Kα radiation *λ* = 1.5406) with the 2θ° ranging from 5 to 80°. FTIR was conducted on an Avatar 320. XPS spectra were collected on an Axis Ultra (Kratos Analytical, UK) XPS spectrometer equipped with an Al Ka source (1486.6 eV). TGA was conducted on a TA instrument Q500 under air atmosphere with a ramping rate of 5 ^o^C min^–1^ from room temperature to 900 ^o^C.

### Electrode fabrication and testing

A conventional slurry-coating process was used to fabricate the electrodes. The active material powders, Super P conductive agent, and poly(vinylidene fluoride) binder were mixed in a mass ratio of 80:10:10, and homogenized in *N*-methyl-2-pyrrolidone to form slurries. The homogenous slurries were uniformly coated on carbon-coated Al foil substrates and dried at 60 ^o^C for 8 h. The mass loading on each electrode was controlled to be 3.0–3.3 mg cm^–2^, corresponding to S mass loading of 1.8–1.9 mg cm^–2^. The Li–S battery performance was tested using 2016-type coin cells with lithium discs as the counter electrodes, Celgard 3501 membrane as the separator, and 1.0 M LITFSI in 1:1 v/v DOL/DME containing LiNO_3_ (2 wt%). A relatively low electrolyte/sulfur ratio of 12:1 µL is applied. CV measurements were carried out on a VSP300 potentiostat/galvanostat (Bio-Logic LLC, Knoxville, TN) using cutoff voltages of 3.0 and 1.5 V versus Li/Li^+^. The galvanostatic charge/discharge measurements were performed on NEWARE BTS-CT3008 (Neware Technology, Ltd, Shenzhen, China) at different current densities. Electrochemical impedance spectroscopy measurement was conducted on a Princeton Applied Research VersaSTAT MC potentiostat. The Nyquist plots were recorded potentiostatically by applying an AC voltage of 10 mV amplitude in the frequency range of 10^5^ to 0.01 Hz. All electrochemical measurements were carried out at room temperature.

### DFT calculation in detail

Vienna ab initio simulation package (VASP)^65^ program was conducted for DFT^66,67^. Core electrons were described by the projector augmented-wave (PAW) pseudopotentials^68,69^, and exchange-correlation energies of electrons used the Perdew, Burke, and Ernzerhof (PBE) functional^70^ for generalized gradient approximation (GGA). The Methfessel–Paxton smearing method were utilized. The cutoff energy for expanded plane wave basis set used 520 eV. All ions were fully relaxed during the structural optimization until the total energy was converged within 10^–4^ eV. The 30 × 30 × 30 Å^3^ unit cell box was used for calculation of single molecule of AQ and Li_2_Sn (4 ≤ *n* < 8) with (1 × 1 × 1) *k*-points mesh. A gamma point mesh with (3 × 6 × 1) and (3 × 3 × 1) *k*-points was used for the graphene unit cell (4√3 × 2√3) and (4√3 × 4√3), respectively. For the slab models, the Brillouin zone with a vacuum space of 28 Å was employed to avoid interactions between top and bottom surface.

### Data availability

All relevant data supporting the findings of this study are available from the authors on request.

## Electronic supplementary material

Supplementary Information
