# Supplementary material for: Chemisorption of polysulfides through redox reactions with organic molecules for lithium–sulfur batteries
Source: Nat Commun. 2018 Feb 16;9:705. doi: 10.1038/s41467-018-03116-z (PMC5816018; doi:10.1038/s41467-018-03116-z)
Supplement: Supplementary file 1 — Supplementary Information [file 41467_2018_3116_MOESM1_ESM.pdf]

Supplementary Figures

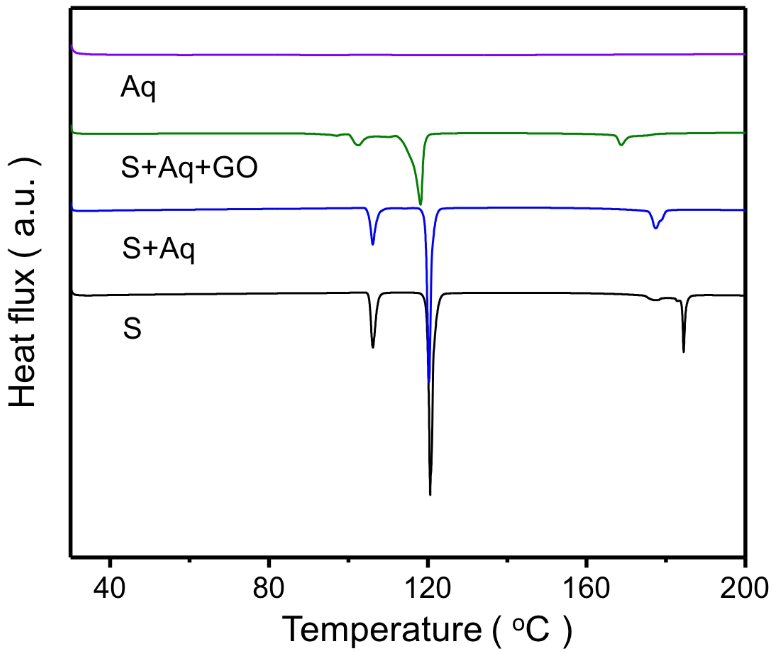

Supplementary Figure 1. DSC curves obtained for pure S, pure AQ, S-AQ and S-AQ-G composites.

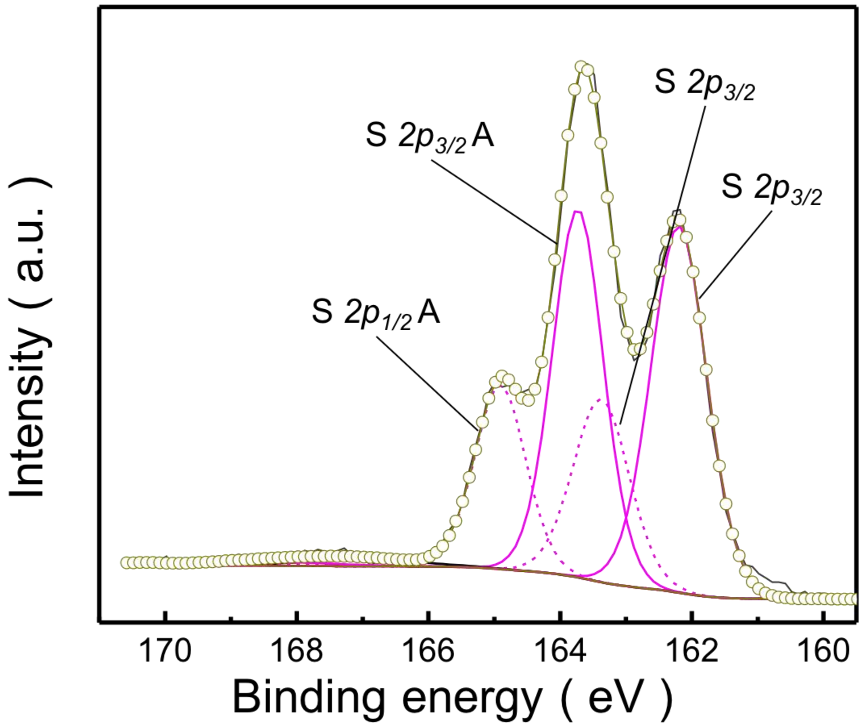

Name	Peak Position (eV)	Atomic Ratio (%)
S *2p* $_{3/2}$	162.20	51.2
S *2p* $_{1/2}$	163.38	
S *2p* $_{3/2}$ A	163.73	48.8
S *2p* $_{1/2}$ A	164.91	

Supplementary Figure 2. XPS core-leveled scan of Li_2S_4 and its quantitative analysis.

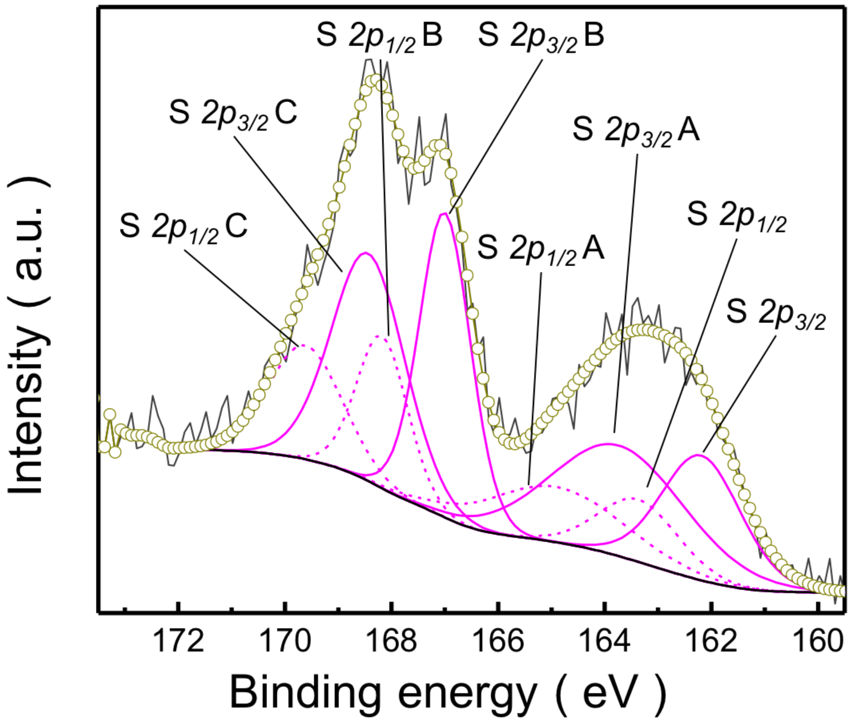

Name	Peak Position (eV)	Atomic Ratio (%)
S *2p* $_{3/2}$	162.19	17.3
S *2p* $_{1/2}$	163.37	
S *2p* $_{3/2}$ A	163.73	26.0
S *2p* $_{1/2}$ A	163.91	
S *2p* $_{3/2}$ B	167.00	27.3
S *2p* $_{1/2}$ B	168.18	
S *2p* $_{3/2}$ C	168.44	29.4
S *2p* $_{1/2}$ A	169.62	

Supplementary Figure 3. XPS core-leveled scan of AQ/Li_2S_4 and its quantitative analysis.

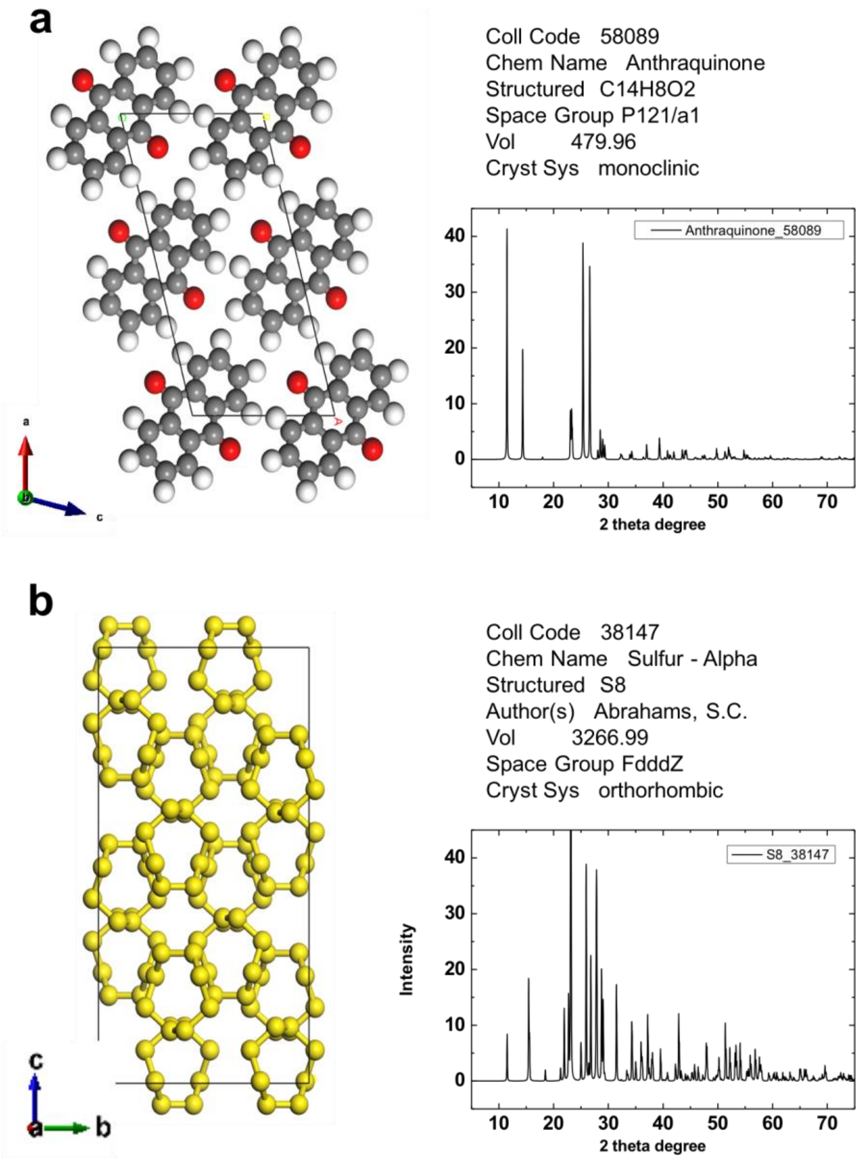

Supplementary Figure 4. Theoretically calculated XRD patterns.

a XRD pattern of AQ and **b** XRD pattern of sulfur from ICSD.

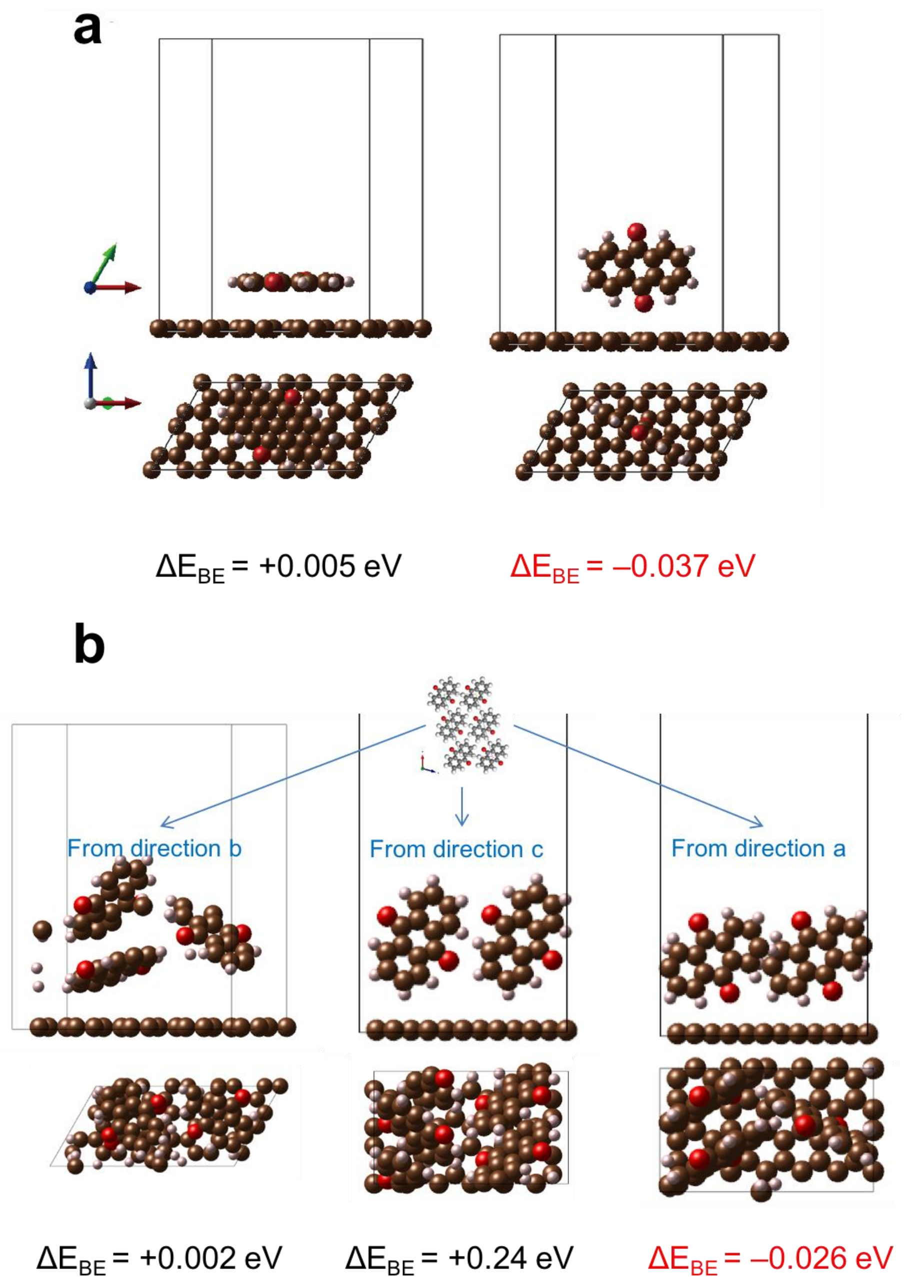

Supplementary Figure 5. Different AQ/G models with different AQ adsorption directions.

a Considered AQ/G models depending on adsorption direction of AQ: along plane and vertical; **b** Monoclinic crystal structures of AQ on graphene designed by different direction from AQ unit cell. Vertical distance of AQ molecules on graphene is reduced compared with those of pure AQ molecules, evaluating at 3.77137 Å and 3.7215 Å for pure AQs and AQs on graphene, respectively. The reduced vertical distance might indicate to stronger π-π interaction.

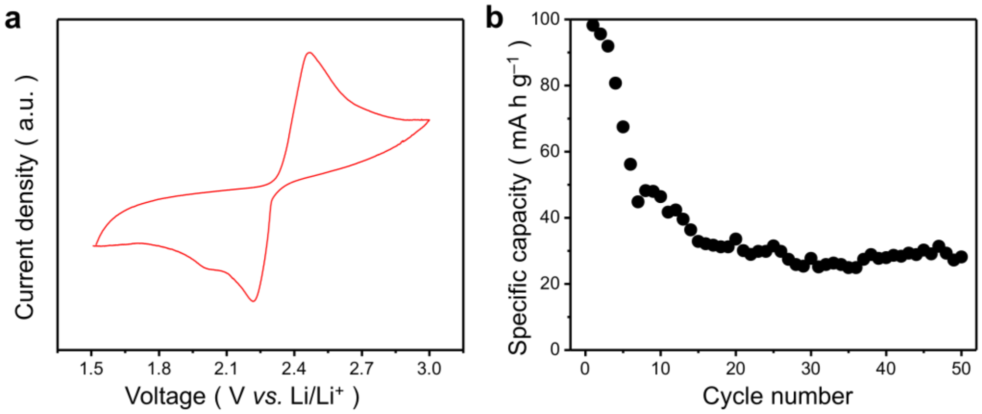

Supplementary Figure 6. Electrochemical property of AQ molecule.

a CV curve of pure AQ at a scan rate of 0.1 mV s^{-1} within the voltage window ranging from 3.0 to 1.5 V *vs.* Li/Li^{+}; **b** Cycling performance of pure AQ at a current density of 50 mA g^{-1} for 50 cycles.

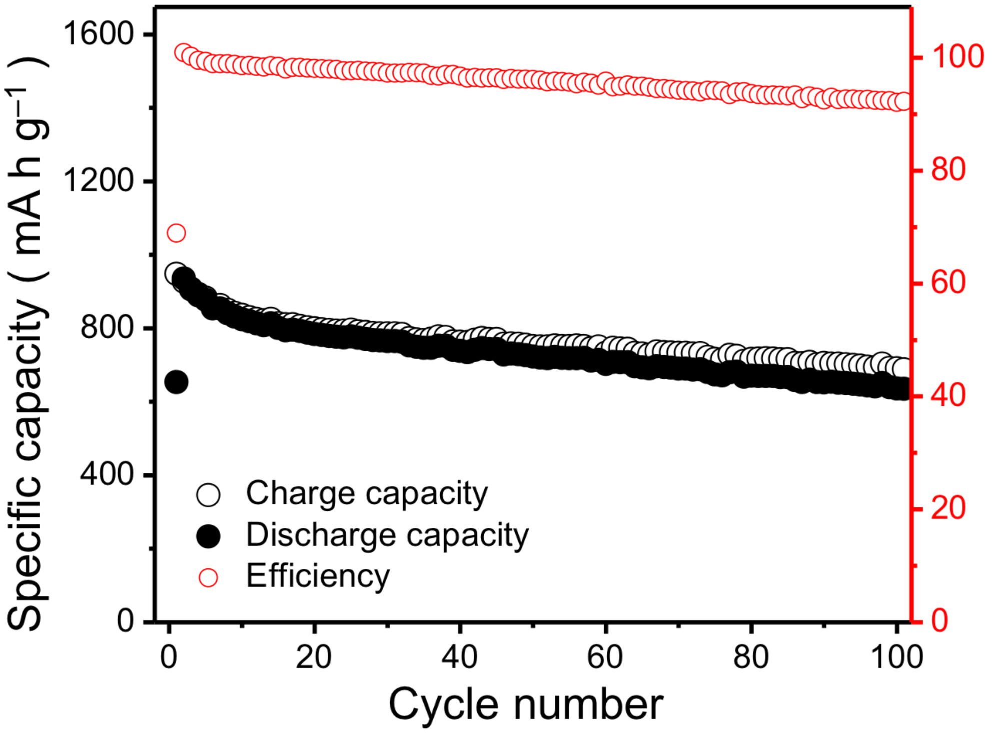

Supplementary Figure 7. Cycling performance of S-G prepared at identical condition and ratio to S-AQ-G at a current density of 0.2 C-rate for 100 cycles.

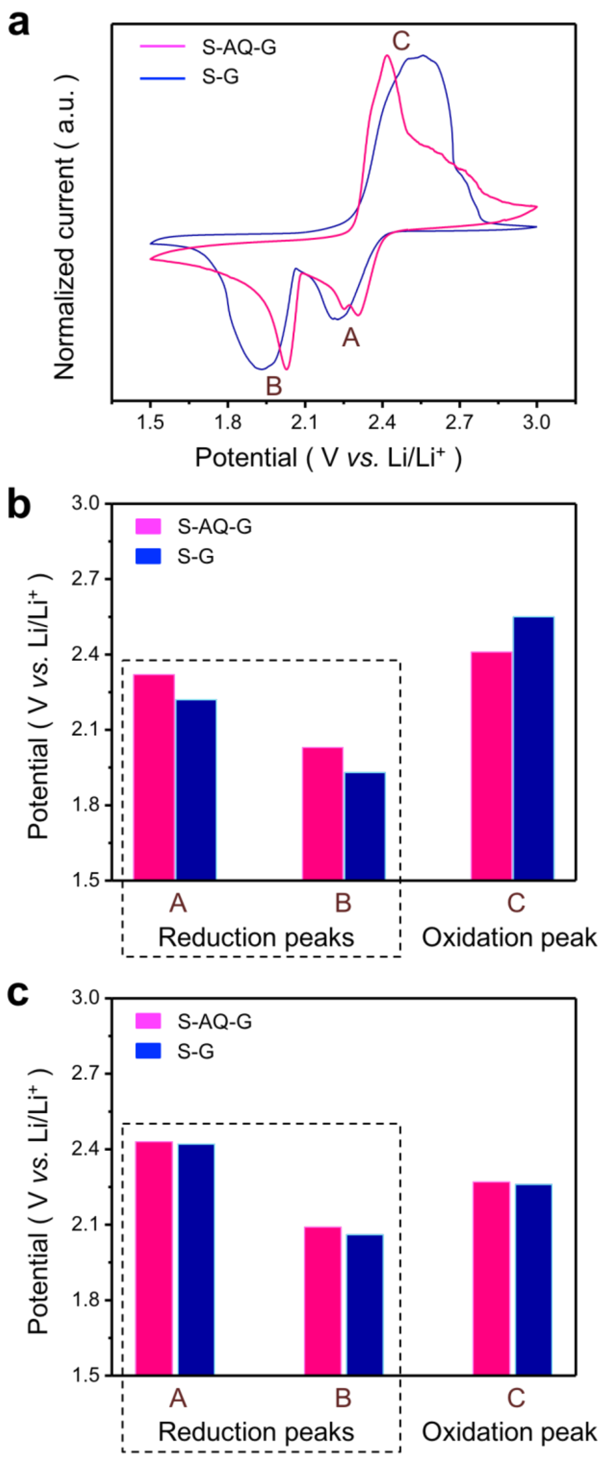

Supplementary Figure 8. Understanding of the kinetics of S-AQ-G composites.

a Comparison of the CV curves of S-AQ-G composites and S-G counterpart at a scan rate of 0.1 mV s^{-1}; and **b, c** the corresponding comparison of peak potentials. Comparison of the onset potentials of S-AQ-G composites and S-G counterpart based on the CV curves at a scan rate of 0.1 mV s^{-1}.
